# Conditioned pain modulation is associated with anesthesia efficacy in patients with temporomandibular disorder

**DOI:** 10.1590/1807-3107bor-2026.vol40.092

**Published:** 2026-11-09

**Authors:** Isadora Alves LORENZO, Lucas Daniel Lago Lopes CUNHA, Allen Matheus da Silva NASCIMENTO, Rhayane da Conceição MONTEIRO, Leonardo Rigoldi BONJARDIM, Yuri Martins COSTA

**Affiliations:** (a) Universidade Estadual de Campinas, Piracicaba Dental School, Department of Biosciences, Piracicaba, SP, Brazil.; (b) Universidade de São Paulo – USP, Bauru School of Dentistry, Department of Biological Sciences, Bauru, SP, Brazil.

**Keywords:** Facial pain, Temporomandibular Joint Disorders, Anesthesia, Chronic Pain

## Abstract

Conditioned pain modulation (CPM) assesses descending pain inhibition in humans and has been shown to be clinically relevant. However, the possible contribution of the CPM response to the efficacy of local anesthetics remains poorly understood. This study aimed to evaluate the association between the CPM response and the efficacy of topical anesthesia applied to the trigeminal region in females with chronic orofacial pain and healthy participants. Thirty-five females with painful temporomandibular disorder (TMD) and twenty healthy females were included. Somatosensory sensitivity of the masseter region was assessed at baseline and after the application of 4% lidocaine and placebo creams in three separate sessions. A sequential CPM protocol was assessed at baseline. The test stimulus (TS) was the pressure pain threshold of the anterior temporalis, and the conditioning stimulus was immersion of the forearm in hot water for 1 minute. CPM magnitude was calculated as the relative change between the unconditioned and conditioned TS, which was correlated with the relative changes in somatosensory sensitivity between the lidocaine/placebo and baseline sessions. The significance level was set at 0.2% following a Bonferroni correction (p = 0.002). There was a moderate, positive correlation (r = 0.54; p = 0.001) between the relative change in the cold detection threshold in the lidocaine session and CPM for participants with chronic painful TMD. There was no correlation between the degree of anesthesia and CPM in healthy females. The efficiency of descending pain inhibition may influence the effectiveness of topical anesthesia in patients with chronic orofacial pain.

## Introduction

Local anesthetics block the transmission of nociceptive impulses, thereby reducing associated nociceptive pain.^1^ Research efforts have mainly focused on enhancing anesthetic efficacy through improvements in formulation, pharmacokinetics, and pharmacodynamics.^2^ However, relatively little attention has been directed toward patient-specific factors that may also influence anesthesia outcomes. For instance, the possible contribution of conditioned pain modulation (CPM) magnitude to local anesthetic response remains underexplored.

CPM refers to the use of psychophysical techniques to clinically assess descending pain inhibition.^3^ This phenomenon was first described in animal models by Le Bars et al.^4^ and termed diffuse noxious inhibitory control (DNIC). These studies showed that the responses of wide dynamic range (WDR) neurons, located in the spinal cord or the spinal trigeminal nucleus, were inhibited by a remote nociceptive conditioning stimulus.^4-6^ In humans, CPM paradigms evaluate DNIC-like effects, where a painful conditioning stimulus reduces the perceived intensity of a test stimulus, although this inhibitory effect is not consistently observed across individuals.^7,8^

The assessment of endogenous pain modulation using the CPM paradigm is clinically relevant. Evidence indicates impaired pain inhibition in various chronic pain conditions.^3,9,10^ In this context, patients with chronic pain who exhibit deficient inhibitory pain modulation may benefit from therapies specifically designed to enhance endogenous inhibitory tone.^9^ Moreover, the magnitude of the CPM inhibitory response has been associated with greater pain reduction following treatment in patients with chronic painful temporomandibular disorders (TMD), such as myalgia.^11^ Moreover, as expected for pain sensitivity assessment in humans, psychological factors such as anxiety, pain catastrophizing and stress can also influence the CPM response.^12,13^

Central mechanisms can either amplify or dampen the incoming nociceptive signal, influencing the behavioral expression of the topical anesthetic’s effect.^14^ For example, experimental evidence shows that nocebo responses, which recruit central facilitatory circuits, can significantly diminish or even reverse the perceived effect of a topical anesthetic.^14^ This does not abolish its peripheral pharmacodynamic action but illustrates how powerful central modulation can be in shaping the pain experience.

In this context, it is physiologically plausible to investigate whether individuals with more effective descending pain inhibition, as assessed by the CPM paradigm, experience greater overall analgesia when peripheral input is concurrently reduced. This hypothesis does not suggest that CPM alters the pharmacological properties of lidocaine; rather, it considers the possibility that endogenous inhibitory tone and peripheral blockade jointly influence the final perceptual outcome. Conversely, individuals with less efficient CPM may show a smaller reduction in pain following topical anesthesia. Supporting this line of reasoning, a recent study in patients with burning mouth syndrome reported that those with minimal pain reduction after a lingual nerve block with lidocaine also exhibited lower CPM efficiency, evaluated at two different body sites, relative to healthy participants.^15^ While preliminary, such findings illustrate the potential relevance of interactions between central modulation and peripheral analgesic responses.

The evaluation of both anesthetic responses and pain modulation may also broaden the understanding of pain mechanisms in patients with chronic pain.^16^ A valuable tool for assessing the efficacy of topical anesthesia is the German battery of quantitative sensory tests (QST), which is widely recognized for its standardization, reliability, and comprehensiveness.^17-19^ This set of psychophysical tests allows the detection of meaningful differences in the degree of hypoalgesia and hypoesthesia following anesthesia;^16,20^

Based on this, our study aimed to investigate the correlation between CPM magnitude and the efficacy of topical anesthesia applied to the trigeminal region in females with chronic painful TMD. Our *a priori* hypothesis was that a significant association exists between CPM magnitude and the anesthetic response.

## Methods

### Sample and ethics

This study was approved by the Research Ethics Committee of the Bauru School of Dentistry, University of Sao Paulo, Bauru, São Paulo, Brazil (registration code: CAAE 21115213.7.0000.5417.). All participants were informed of the research purposes and signed an informed consent form.

This study was designed as an exploratory investigation, with the sample size determined based on previous studies.^16^ The sample consisted of females seeking dental care for facial pain at the Bauru School of Dentistry, University of São Paulo, and female staff and students. Participants were recruited by convenience sampling. The study included 35 females with chronic painful TMD and 20 healthy females.

Inclusion criteria for the TMD group were: a) female, aged over 18 years; b) diagnosis of painful TMD according to the Diagnostic Criteria for Temporomandibular Disorders (DC/TMD); and c) pain duration of at least 3 months. For healthy participants, inclusion criteria were: a) female, aged over 18 years; and b) good systemic health. Exclusion criteria for both groups were: a) history of head trauma; b) presence of orofacial pain in the previous 30 days (for healthy participants only); c) serious medical conditions; d) regular use of medications, defined as daily or habitual intake of drugs such as antidepressants, anticonvulsants, or nonsteroidal anti-inflammatory drugs; e) psychiatric disorders; f) other chronic regional or generalized pain conditions such as headaches, low back pain, or fibromyalgia; and g) prior treatment for painful TMD within the last 3 months. Eligibility was assessed through detailed interviews. All participants were instructed to avoid analgesic use for 24 hours prior to assessments.

Diagnosis of painful TMD was established using the DC/TMD. To exclude temporomandibular disorders in healthy participants, the TMD pain screening questionnaire was administered.^21^

### Experimental design

The study consisted of three sessions, each separated by 24 hours (Figure 1). Participants received a 30-minute application of 4% lidocaine (Dermomax^®^, Aché Laboratórios Farmacêuticos AS, Guarulhos, Brazil) in one session and a placebo cream in another. Both creams were applied topically in a standardized amount (7 ± 3 g) to cover the skin of the entire anatomical area of the most painful masseter muscle, based on self-report and/or clinical examination for TMD patients, or the right masseter muscle for healthy participants. The order of cream administration was randomized by block (two blocks: TMD and healthy participants) using a computer-generated randomization list for each participant (http://www.randomization.com). Both participants and the examiner were blinded to the cream content (double-blind design). Somatosensory variables were assessed in all three sessions, at baseline and immediately after the application of either lidocaine or placebo cream. CPM, psychosocial questionnaires, and clinical characteristics were assessed only at baseline.

**Figure 1:**
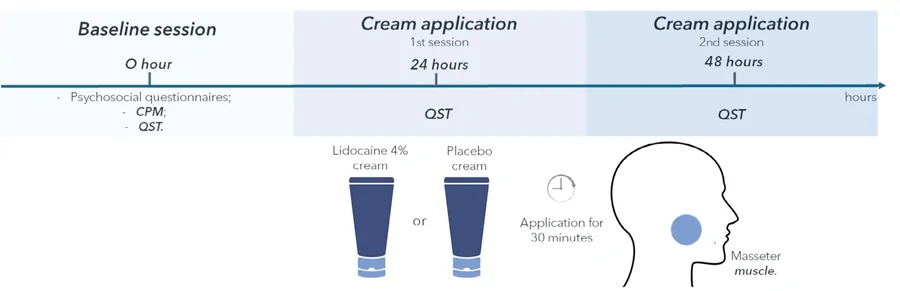
Study design.

The examiner was not present during cream application. Additionally, the person responsible for the randomization process and cream application was not involved in data collection or analysis.

### Outcomes

The primary outcomes for this study were: a) relative changes in somatosensory thresholds between the baseline session and the lidocaine or placebo sessions; and b) CPM magnitude. Psychosocial questionnaires and clinical characteristics were considered secondary variables. Somatosensory assessments were conducted according to the recommendations and guidelines of the DFNS quantitative sensory testing protocol. The following QST parameters were evaluated: cold detection threshold (CDT), warm detection threshold (WDT), cold pain threshold (CPT), heat pain threshold (HPT), mechanical detection threshold (MDT), mechanical pain threshold (MPT), wind-up ratio (WUR), and pressure pain threshold (PPT).^18,19^

#### Somatosensory assessment

CDT, WDT, CPT, and HPT were assessed using the TSA 2001-II computerized thermal stimulator (MEDOC, Ramat Yishai, Israel) with a probe contact area of 2.56 cm^2^. Each threshold was determined as the mean of three consecutive measurements under the following settings: basal temperature of 32°C, stimulus ramp of 1°C/s, and temperature thresholds of 0°C and 50°C.^18^

MDT was assessed using a standardized set of Semmes-Weinstein monofilaments (Touch-TestTM Sensory Evaluators, North Coast Medical Inc, Gilroy, USA), which apply forces between 0.008 g/mm^2^ to 300 g/mm^2^. MPT, was assessed using pointed-tip stimulators (PinPrick Stimulator Set, MRC Systems GmbH, Heidelberg, Germany) with a contact area of 0.2 mm in diameter and calibrated forces ranging from 8 to 512 mN. Participants reported the first sensation of touch (MDT) or pain (MPT), following a series of ascending and descending stimulus intensities.^22,23^ The geometric mean of the three trials was calculated.^18,19^

WUR was assessed using the same set of pointed-tip stimulators. For each participant, the pointed-tip stimulator perceived as “slightly painful” was selected for the test.^18,19^ Pain intensity was rated on a 0–10 numerical scale after a single “pinprick” stimulus and after a series of ten stimuli within a 1 cm^2^ area at 1 Hz. WUR was calculated by dividing the mean pain rating of the three series of ten stimuli by the mean rating of the three individual stimuli.^18,19^

PPT was measured with a digital algometer (DDK20, Kratos Ltda, Cotia, Brazil) fitted with a flat, circular tip of 1 cm^2^, applying pressure to the masseter muscle at a rate of approximately 0.5 kgf/cm^2^/s. Participants were instructed to press a button at the first painful sensation and informed that the objective was not to measure pain tolerance. PPT was calculated as the average of three measurements.^18,19^

#### Conditioned pain modulation

The magnitude of descending pain inhibition was assessed using the following protocol: the test stimulus (TS) consisted of the PPT measured on the anterior region of the temporalis muscle on the painful side (TMD group) or on the right side (control group). The conditioning stimulus (CS) involved immersing the participant’s non-dominant hand in a water bath maintained at approximately 45°C for 1 min. The protocol was sequential, with the TS repeated immediately after CS application. CPM magnitude was calculated as the relative difference between the “TS before the CS” and the “TS after the CS”, with negative values indicating a more efficient CPM effect.^3,24^

#### Psychosocial and clinical assessment

Psychosocial data were collected using validated Brazilian Portuguese translations of self-report questionnaires: the Pittsburgh Sleep Quality Index (PSQI),^25^ the Pain-Related Self-Statement Scale’s Catastrophizing Thoughts subscale (PRSS-C)^26^ and the Hospital Anxiety and Depression Scale (HADS).^27^ Additionally, facial pain intensity reported by TMD patients (average pain during the past week) was assessed using a 0–10 numerical rating scale (0 = no pain and 10 = worst pain imaginable).

## Statistics

Relative differences between the baseline session and the lidocaine or placebo sessions for the somatosensory assessment were calculated using the formula: (X / Y - 1) * 100, where X represents the QST values in the lidocaine or placebo session, and Y represents the baseline values. The formula was adjusted for each QST parameter so that negative values indicated hypoalgesia or hypoesthesia. Normality of the data was assessed using the Kolmogorov-Smirnov test (significance level of 5%, p = 0.050) and by graphical analysis of the Q-Q plots.

Relative changes are presented as mean and standard deviation (SD), standard error of the mean, and 95% confidence interval (95%CI) of the mean. Psychosocial variables were described using either mean and SD or median and interquartile range, as appropriate. Other quantitative variables, such as age and pain intensity, were also reported as mean and SD.

Pearson correlation coefficients, or the nonparametric Spearman coefficient when indicated, were calculated to measure the linear association between a) relative differences in somatosensory parameters and CPM, b) psychosocial measures, and between c) CPM and d) psychosocial measures.

The effect size of the coefficient was classified as small (< 0.1), medium (0.1–0.5), or strong (> 0.5). Finally, given the multiple comparisons, the significance level was adjusted using the Bonferroni correction. Four families of comparisons were defined based on the preferentially assessed nerve fibers for somatosensory assessment: A-δ fibers (CDT, MPT, and WUR); C fibers, (CPT, WDT, HPT, and PPT); and A-β fibers (MDT), in addition to psychosocial assessments. These families of comparisons were analyzed separately for each session (lidocaine and placebo) and group (females with painful TMD and healthy participants). Moreover, we included the associations between CPM and the psychosocial measures as two additional families. Thus, the significance level adopted following the Bonferroni correction was 0.2% (p = 0.002).

Additional statistical analyses were conducted to compare CPM responses and psychosocial characteristics between females with painful TMD and healthy participants. For these comparisons, the independent samples t-test or its nonparametric equivalent, the Mann-Whitney test, was applied as appropriate.

## Results

The mean (SD) age of participants with chronic painful TMD and healthy participants was 34.6 (11.6) and 30.2 (7.7) years, respectively. Participants with chronic painful TMD reported poorer sleep quality, as well as higher levels of pain catastrophizing, anxiety, and depression when compared to healthy participants (Table 1). In addition, participants with chronic painful TMD reported a mean (SD) pain intensity of 6.55 (1.82) and a mean (SD) pain duration of 75.4 (80.7) months.

**Table 1:** Psychosocial characteristics of participants with chronic painful temporomandibular disorder (TMD) and healthy participants.

Variables	Healthy	TMD	p-value
Pain catastrophizing*	0.22 (0.44)	1.44 (1.78)	< 0.001
Symptoms of anxiety	4.5 (2.87)	8.54 (3.79)	< 0.001
Symptoms of depression	2.2 (1.3)	5.0 (3.1)	< 0.001
Sleep quality	5.1 (2.57)	7.89 (3.76)	0.005

*Description provided by the median and interquartile range. For the other variables, the description was based on the mean and standard deviation.

The CPM effect in participants with chronic painful TMD was significantly less efficient compared with healthy females (p = 0.004). The mean (SD) CPM magnitude was -28.45% (29.7) with a 95%CI: -38.6 to -18.2% for TMD patients, and -56.1% (38.0) with a 95%CI: -73.2 to -38.2% for the healthy females.

A moderate positive correlation (r = 0.54; 95%CI: 0.22–0.74; p = 0.001) was observed between the relative change in CDT during the lidocaine session and CPM magnitude among participants with chronic painful TMD (Figure 2A). On the other hand, no significant associations were found between relative changes in the other QST parameters during the lidocaine session for participants with chronic painful TMD (Figure 2A–C). Similarly, no significant correlations were observed for somatosensory changes in the placebo session for participants with chronic painful TMD (Figure 2A–C). In healthy participants, CPM magnitude was not significantly associated with relative changes in QST parameters during either the lidocaine or placebo sessions (Figure 3A–C).

**Figure 2:**
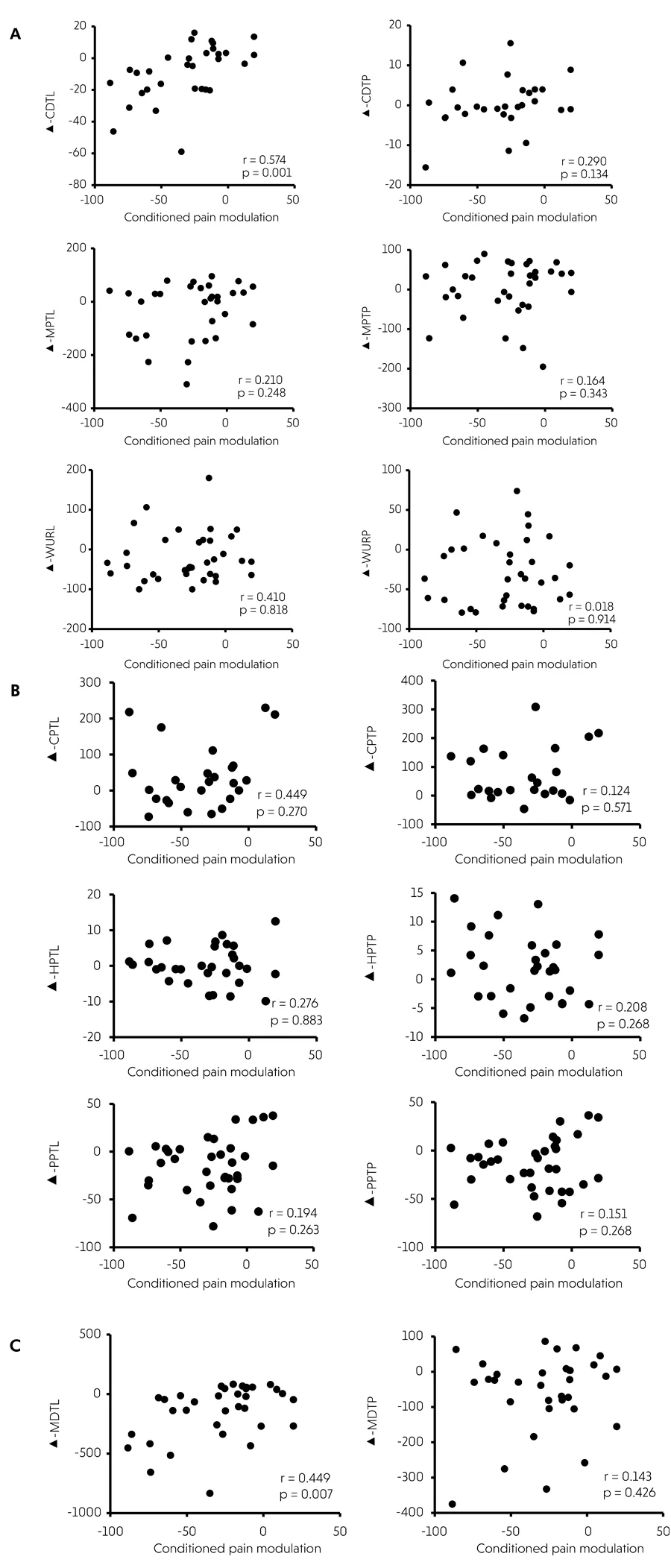
Correlations between the relative changes in somatosensory test outcomes and the magnitude of conditioned pain modulation (CPM) in participants with painful temporomandibular disorder (TMD).

(A) Somatosensory tests that preferentially activate Aδ fibers, (B) somatosensory tests that preferentially activate C fibers, and (C) somatosensory tests that preferentially activate Aβ fibers. CDTL and CDTP = relative change between baseline and lidocaine (L) or placebo (P) sessions for cold detection threshold. MDTL and MDTP: relative change between baseline and lidocaine (L) or placebo (P) sessions for mechanical detection threshold; PPTL and PPTP: relative change between baseline and lidocaine (L) or placebo (P) sessions for pressure pain threshold; MPTL and MPTP: relative change between baseline and lidocaine (L) or placebo (P) sessions for mechanical pain threshold; WDTL and WDTP: relative change between baseline and lidocaine (L) or placebo (P) sessions for warm detection threshold. CPTL and CPTP: relative change between baseline and lidocaine (L) or placebo (P) sessions for cold pain threshold; HPTL and HPTP: relative change between baseline and lidocaine (L) or placebo (P) sessions for heat pain threshold; WURL and WURP: relative change between baseline and lidocaine (L) or placebo (P) sessions for temporal summation ratio.

**Figure 3:**
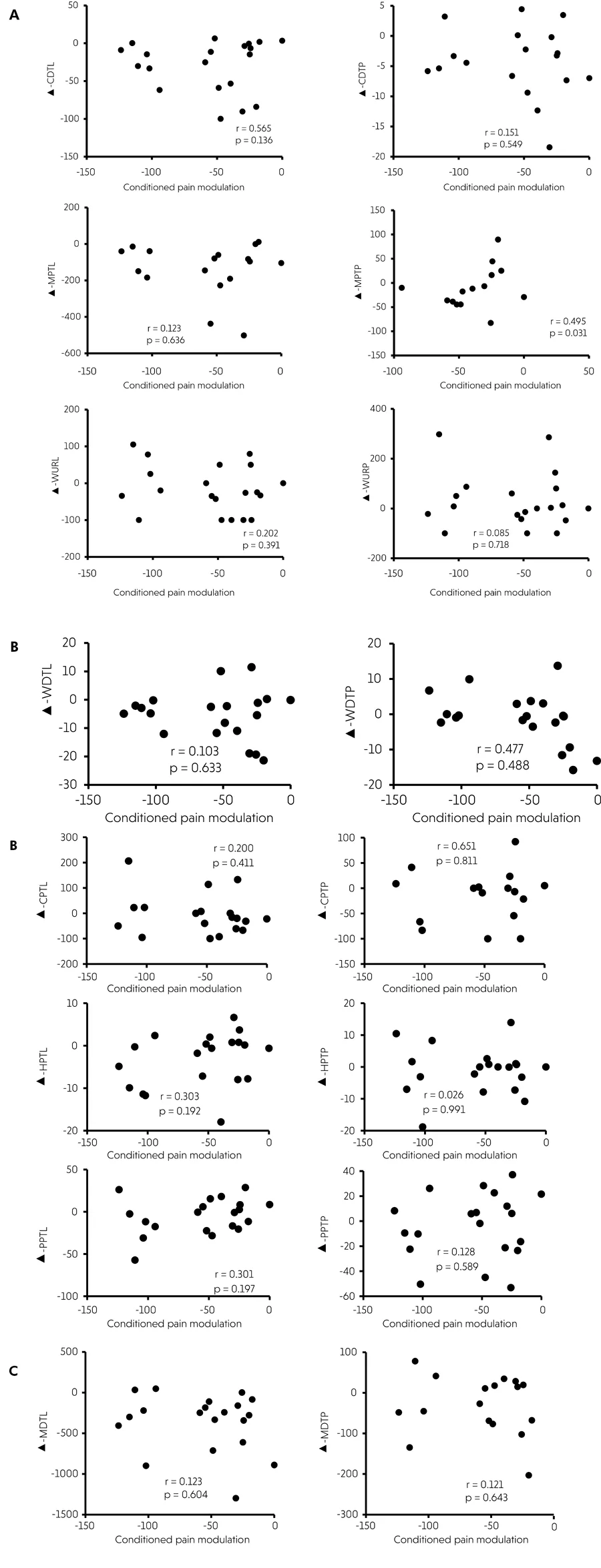
Correlations between the relative changes in somatosensory test outcomes and the magnitude of conditioned pain modulation (CPM) in healthy participants.

(A) Somatosensory tests that preferentially activate Aδ fibers, (B) somatosensory tests that preferentially activate C fibers, and (C) somatosensory tests that preferentially activate Aβ fibers. CDTL and CDTP = relative change between baseline and lidocaine (L) or placebo (P) sessions for cold detection threshold. MDTL and MDTP: relative change between baseline and lidocaine (L) or placebo (P) sessions for mechanical detection threshold; PPTL and PPTP: relative change between baseline and lidocaine (L) or placebo (P) sessions for pressure pain threshold; MPTL and MPTP:= relative change between baseline and lidocaine (L) or placebo (P) sessions for mechanical pain threshold; WDTL and WDTP: relative change between baseline and lidocaine (L) or placebo (P) sessions for warm detection threshold. CPTL and CPTP = relative change between baseline and lidocaine (L) or placebo (P) sessions for cold pain threshold; HPTL and HPTP: relative change between baseline and lidocaine (L) or placebo (P) sessions for heat pain threshold; WURL and WURP: relative change between baseline and lidocaine (L) or placebo (P) sessions for temporal summation ratio.

No significant correlations were observed between psychosocial factors and CPM magnitude in either participants with painful TMD or healthy participants (p > 0.002, Figure 4). Additionally, no significant correlations were found between the relative changes in QST parameters and psychosocial variables in participants with painful TMD or healthy participants (p > 0.002; Figures 5 and 6).

**Figure 4:**
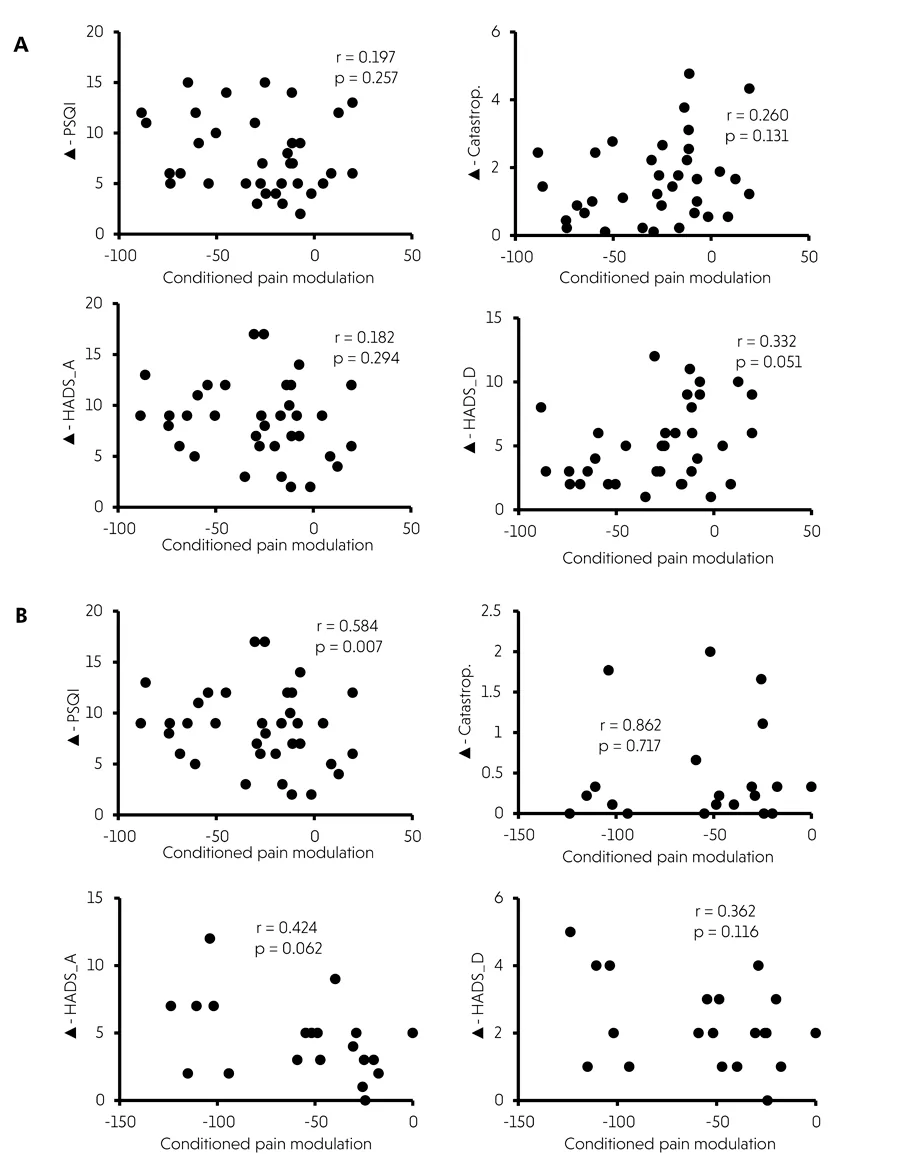
Correlations between the relative changes in psychosocial test outcomes and the magnitude of conditioned pain modulation (CPM) in the painful temporomandibular disorder (TMD) group (A) and in the healthy participants (B).

**Figure 5:**
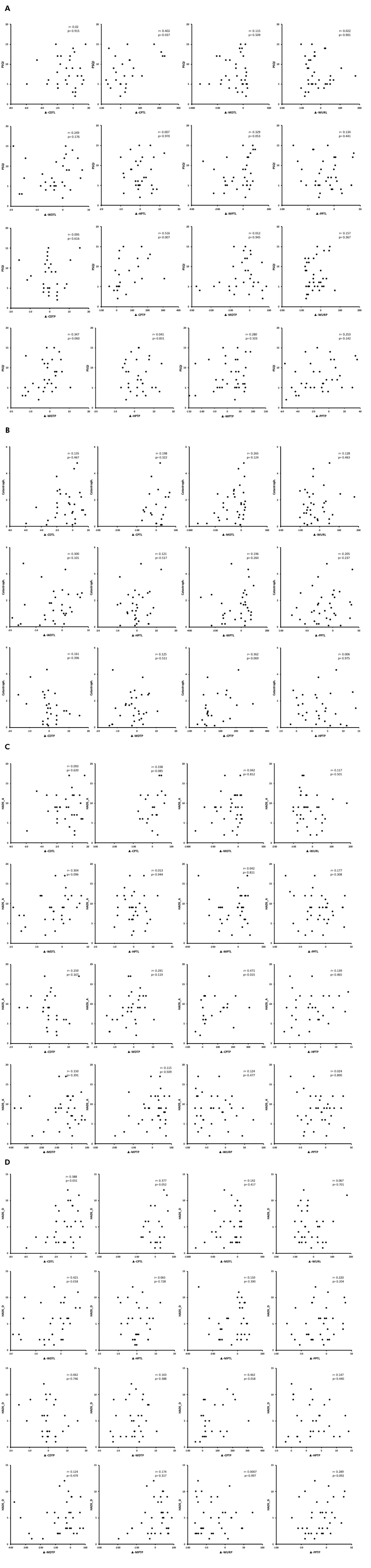
Correlations between relative changes in somatosensory test outcomes and psychosocial variables in participants with painful temporomandibular disorder (TMD).

(A) Pittsburgh Sleep Quality Index (PSQI), (B) Pain catastrophizing (Catastroph.), (C) Anxiety (HADS_A), and (D) Depression (HADS_D). CDTL and CDTP: relative change between baseline and lidocaine (L) or placebo (P) sessions for cold detection threshold. MDTL and MDTP = relative change between baseline and lidocaine (L) or placebo (P) sessions for mechanical detection threshold; PPTL and PPTP: relative change between baseline and lidocaine (L) or placebo (P) sessions for pressure pain threshold; MPTL and MPTP: relative change between baseline and lidocaine (L) or placebo (P) sessions for mechanical pain threshold; WDTL and WDTP: relative change between baseline and lidocaine (L) or placebo (P) sessions for warm detection threshold; CPTL and CPTP: relative change between baseline and lidocaine (L) or placebo (P) sessions for cold pain threshold; HPTL and HPTP: relative change between baseline and lidocaine (L) or placebo (P) sessions for heat pain threshold; WURL and WURP: relative change between baseline and lidocaine (L) or placebo (P) sessions for temporal summation ratio.

**Figure 6:**
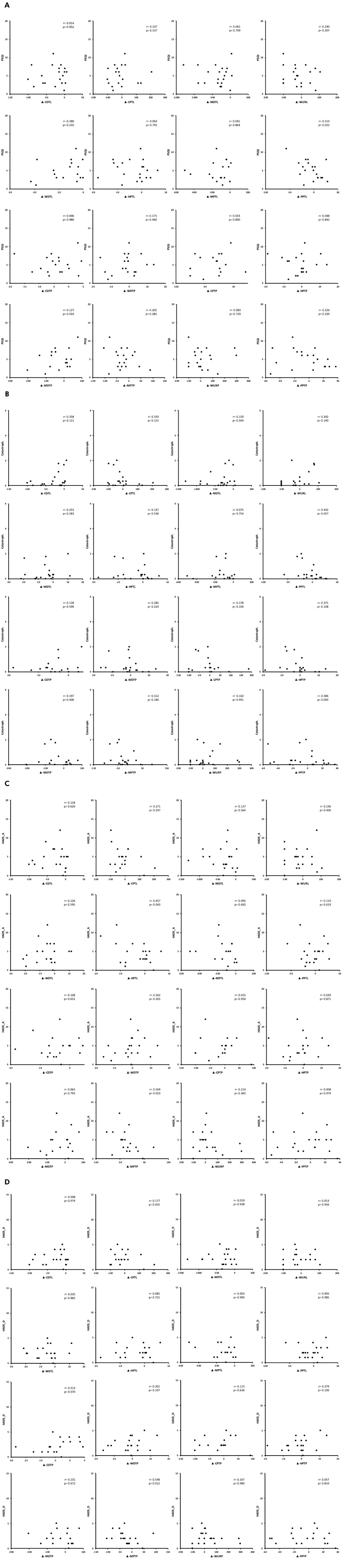
Correlations between relative changes in somatosensory test outcomes and psychosocial variables in healthy participants.

(A) Pittsburgh Sleep Quality Index (PSQI), (B) Pain catastrophizing (Catastroph.), (C) Anxiety (HADS_A), and (D) Depression (HADS_D). CDTL and CDTP: relative change between baseline and lidocaine (L) or placebo (P) sessions for cold detection threshold; MDTL and MDTP: relative change between baseline and lidocaine (L) or placebo (P) sessions for mechanical detection threshold; PPTL and PPTP: relative change between baseline and lidocaine (L) or placebo (P) sessions for pressure pain threshold; MPTL and MPTP: relative change between baseline and lidocaine (L) or placebo (P) sessions for mechanical pain threshold; WDTL and WDTP: relative change between baseline and lidocaine (L) or placebo (P) sessions for warm detection threshold; CPTL and CPTP: relative change between baseline and lidocaine (L) or placebo (P) sessions for cold pain threshold; HPTL and HPTP: relative change between baseline and lidocaine (L) or placebo (P) sessions for heat pain threshold; WURL and WURP: relative change between baseline and lidocaine (L) or placebo (P) sessions for temporal summation ratio.

Overall, lidocaine did not cause a significant reduction in somatosensory sensitivity in the masseter region of participants with painful TMD. Although relative differences suggest that MDT and MPT increased after lidocaine, this effect was not significantly different from placebo in TMD patients due to the large overlap in the 95% CI of the mean (Table 2). In contrast, relative values indicate hypoalgesia and somatosensory hypoesthesia in healthy participants (Table 3).

**Table 2:** Mean and standard deviation (SD) of quantitative sensory testing (QST) relative differences from baseline of participants with painful temporomandibular disorder (TMD) for both the lidocaine and placebo creams.

TMD	Lidocaine 4%		Placebo	
	Mean (SD) %	95%CI of the Mean	Mean (SD) %	95%CI of the Mean
CDT	-9.06 (17.51)	-15.4 to -2.64	0.25 (6.29)	-2.18 to 2.69
WDT	-3.28 (7.10)	-5.94 to -0.63	-1.85 (6.22)	-4.18 to 0.46
CPT	36.98 (86.90)	1.91 to 72.03	73.78 (91.29)	34.30 to 113.25
HPT	0.19 (5.38)	-1.78 to 2.17	2.00 (5.65)	-0.10 to 4.11
MDT	-151.0 (230.7)	-231.5 to -70.53	-59.86 (113.7)	-100.18 to 19.54
MPT	-31.10 (105.2)	-69.03 to 6.82	2.91 (68.48)	-20.61 to 26.43
WUR	-24.76 (51.84)	-42.88 to -6.63	28.67 (41.71)	-42.93 to -14.27
PPT	-15.36 (29.65)	-25.54 to -5.17	-14.05 (26.16)	-23.04 to -5.06

CDT: cold detection threshold; WDT: warm detection threshold; CPT: cold pain threshold; HPT: heat pain threshold; MDT: mechanical detection threshold; MPT: mechanical pain threshold; WUR: wind up ratio; PPT: pressure pain threshold.

**Table 3:** Mean and standard deviation (SD) of quantitative sensory testing (QST) relative differences from baseline of healthy participants for both the lidocaine and placebo creams.

Healthy	Lidocaine 4%		Placebo	
	Mean (SD) %	95%CI of the Mean	Mean (SD) %	95%CI of the Mean
CDT	-29.35 (33.71)	-45.1 to -13.5	-4.30 (5.70)	-7.14 to -1.47
WDT	-5.34 (8.64)	-9.3 to -1.2	-1.15 (7.29)	-4.56 to 2.26
CPT	-4.63 (80.18)	-43.2 to -34	-16.78 (52.42)	-44.71 to 11.15
HPT	-3.26 (6.27)	-6.2 to -0.33	-1.03 (7.32)	-4.46 to 2.39
MDT	-362.61(352.48)	-527.5 to -197.6	-31.16 (72.74)	-68.56 to 6.24
MPT	-137.84 (142.82)	-211.2 to – 64.4	-22.93 (45.47)	-44.85 to -1.01
WUR	-16.45 (65.15)	-46.9 to 14.4	28.67 (110.10)	-22.58 to 80.20
PPT	-5.40 (21.14)	-15.2 to 4.49	-3.96 (26.35)	-16.30 to 8.36

CDT: cold detection threshold; WDT: warm detection threshold; CPT: cold pain threshold; HPT: heat pain threshold; MDT: mechanical detection threshold; MPT: mechanical pain threshold; WUR: wind up ratio; PPT: pressure pain threshold.

## Discussion

This study investigated the association between CPM magnitude and the efficacy of topical anesthesia applied to the trigeminal region in patients with chronic painful TMD and healthy participants. The main findings were as follows: a) the descending inhibitory response showed a moderate and significant association with the degree of topical anesthesia in A-δ fibers in patients with chronic painful TMD; b) no association was observed between CPM responses and anesthetic efficacy in healthy participants, and no significant associations were observed between endogenous inhibitory pain modulation and psychosocial assessment.

The significant association between the degree of anesthesia of A-δ fibers and CPM is noteworthy, as it suggests an influence of the descending inhibitory system on the perceptual response to the anesthetic effect. CDT is a somatosensory parameter that predominantly assesses A-δ fibers, which are the fiber type most easily blocked by lidocaine.^28,29^ A previous study, for example, reported that topical lidocaine applied to the masseter produced a partial and selective blockade of A-δ and C fibers,^30^ leading to increased thermal detection thresholds (CDT and WDT), increased MPT, and reduced sensitivity to suprathreshold painful stimuli.^31^ This observation is supported by preclinical evidence indicating that nerve fibers differ in their susceptibility to local anesthetics,^30^ with A-δ fibers appearing to be the most sensitive to somatosensory anesthetic blockade, which can occur at lower concentrations.^29,31^ This differential susceptibility may help explain the absence of correlations between CPM magnitude and psychophysical tests preferentially stimulating A-β and C fibers.

However, this result should be interpreted with caution, as it was not consistently observed across all A-δ-related somatosensory tests. The other two parameters, MPT and WUR, did not show significant changes. The underlying reasons remain unclear but may be related to the inherent variability of MPT and WUR responses. Nevertheless, it is important to emphasize that we considered the issue of multiple comparisons by applying the Bonferroni correction to reduce the chances of type I error, thereby reducing the likelihood of a false positive.^32^

The absence of a significant difference between lidocaine and placebo in the TMD group, which reflects an overall comparison between the interventions, was also an interesting result. This finding is consistent with previous evidence suggesting that individuals with chronic pain may exhibit attenuated peripheral responses to topical anesthesia.^33-35^ However, overall group-level effects do not preclude meaningful individual variability in response. It is possible, and our data suggest, that some patients experienced a relevant sensory loss after lidocaine compared to placebo, but the large variability diluted the group mean so that no significant difference emerged. Thus, the absence of a group-level difference does not contradict the observed correlation. Rather, it suggests that within the heterogeneous TMD sample, patients with more efficient descending pain inhibition demonstrated greater increases in sensory thresholds (in this case, CDT) after lidocaine.

Topical anesthesia efficacy is known to be influenced by substantial interindividual variability in somatosensory sensitivity.^16,30^ Traditionally, heterogeneity in the effects of topical anesthesia has been attributed primarily to pharmacokinetic and pharmacodynamic factors, as well as to structural and functional characteristics of the peripheral nervous system, such as the density and depth of sensory receptors.^2^ In contrast, our results suggest that endogenous pain modulation is a relevant factor to consider when assessing the effects of anesthetics on somatosensory sensitivity. Specifically, our findings indicate that females with chronic painful TMD and less efficient inhibitory CPM may experience reduced local analgesic efficacy. This indicates that endogenous inhibitory tone and peripheral blockade may act synergistically, resulting in a greater reduction in perceived pain in individuals with more efficient CPM, for example.

On the other hand, it is noteworthy that the association between CPM responses and the efficacy of topical anesthesia was not observed in healthy participants. The stronger inhibitory CPM response observed in healthy participants compared with those with painful TMD may partly explain the absence of a significant linear association. Additionally, the magnitude of the anesthetic effect in healthy participants should be considered. A previous study reported that healthy individuals experienced a substantial loss of somatosensory function following topical anesthesia application.^22^ Thus, it is possible that the more robust anesthetic effect masked any potential influence of CPM responses in this group. Additional investigations with larger samples and techniques that allow greater variability in anesthesia levels are recommended to investigate this association more systematically in healthy participants.

Psychosocial factors are considered parameters that account for part of the interpersonal variability in pain perception.^12^ Recent studies suggest that individuals with chronic pain disorders tend to exhibit more depressive symptoms, greater pain catastrophizing, lower quality of life, and inefficient CPM.^36^ However, in the present study, no significant linear associations were found between endogenous inhibitory pain modulation and psychosocial measures. Similarly, no significant correlations were identified between the relative changes in somatosensory test outcomes and psychosocial variables, either in participants with painful temporomandibular disorder (TMD) or in healthy participants. Given the large number of correlations tested, the Bonferroni correction was applied. Thus, these secondary findings do not allow us to definitively rule out any influence of psychosocial factors. However, they make it less likely that the observed association between CPM and the sensory response to lidocaine is merely a by-product of psychosocial aspects of the sample. Moreover, our sample size is too small to support a reliable regression model including CPM and several psychosocial predictors without a substantial risk of overfitting and unstable estimates.

It is also important to highlight that the presence of psychosocial alterations and impaired endogenous pain modulation does not necessarily imply a correlation between them, even though overlapping mechanisms, such as the regulation of serotonin and norepinephrine neurotransmitters, have been described.^12,37^ Furthermore, the association between psychosocial functioning and the CPM response may be non-linear. This suggests that investigations with larger samples and more sophisticated statistical models may be required to clarify what appears to be a small effect size. Additionally, methodological factors related to CPM assessment may also contribute to the observed variability.^3^

CPM has been considered a promising tool to support the prevention, investigation, and management of chronic pain.^38^ Our findings may contribute to this broader context by suggesting a potential influence of descending pain modulation on the sensory response to topical anesthesia. However, this interpretation should be made with caution. The exploratory design, limited evidence in the literature, and the moderate association restricted to a single parameter do not yet allow for definitive conclusions about the clinical relevance of this relationship.

Although the present study does not propose an immediate clinical application, it helps clarify the potential value of integrating CPM-based information into future diagnostic or phenotyping frameworks. In this sense, our findings help strengthen the theoretical foundation necessary for the eventual development of clinically applicable tools derived from the CPM paradigm. As an exploratory investigation, the study also generates data-driven hypotheses regarding how individual differences in endogenous pain modulation may shape sensory responses to local anesthetics, an area that has received limited attention to date.

This exploratory study has additional limitations that should be considered. The sample size was based on a previous study and included only females.^16^ However, this group represents the population most affected by TMD, particularly myalgias, such as the one examined in this study. Epidemiological evidence consistently shows a greater incidence and prevalence,^39,40^ and an increased likelihood of chronicity among females.^41^ Moreover, the non-significant findings in healthy participants may be attributed to a type II error. Drawing definitive conclusions is challenging without larger sample sizes, which would allow for a clearer understanding of the observed effects. Our sample size was too small to support reliable regression models without a substantial risk of overfitting and unstable estimates. Thus, future studies are necessary to confirm our findings. Another limitation relates to the anesthetic technique used. To date, no studies have evaluated the influence of CPM responses on the efficacy of local anesthesia administered via infiltrative techniques or nerve blocks. Consequently, caution is warranted when generalizing the impact of CPM on anesthesia efficacy to other anesthetic modalities.

## Conclusions

The efficiency of descending pain inhibition may influence the efficacy of topical anesthesia targeting A-δ fibers in patients with chronic orofacial pain. These preliminary findings suggest that endogenous pain inhibition can be considered when evaluating anesthetic efficacy or employing local anesthesia as an experimental model in individuals with chronic orofacial pain.

## Data Availability

The datasets generated during and/or analyzed during the current study are available from the corresponding author on reasonable request.
